# Cupping Therapy as a Potential Complimentary Treatment for Meniere's Disease: A Case Report

**DOI:** 10.7759/cureus.55864

**Published:** 2024-03-09

**Authors:** Tariq S Jamal, Khaled S Aseri, Faisal S Alghamdi, Abdullah M Asiri, Abdulrahman S Hakami

**Affiliations:** 1 Otorhinolaryngology Department, Dr. Soliman Fakeeh Hospital, Jeddah, SAU; 2 Community and Preventive Medicine Department, King Abdulaziz Medical City, Jeddah, SAU; 3 Medical School, King Saud bin Abdulaziz University for Health Sciences, Jeddah, SAU; 4 Medical School, Ibn Sina National College for Medical Studies, Jeddah, SAU

**Keywords:** meniere's disease, vertigo, tinnitus, sensorineural hearing loss, cupping therapy, case report

## Abstract

Meniere's disease is defined by the presence of three essential symptoms: episodic vertigo, tinnitus, and sensorineural hearing loss. The mainstay of its management constitutes lifestyle modification and medical and surgical therapies. Cupping therapy is an ancient treatment that is still widely used especially in the Middle East, Africa, and the United Kingdom. This study portraits the case of a 54-year-old patient suffering from long-standing Meniere's disease. The patient was treated with the routine treatment that was to no avail. It was decided that the patient undergoes cupping therapy. Over two years of monthly cupping therapy sessions, the patient reported a decrease in intensity and frequency of symptoms until its disappearance. Cupping therapy has shown a positive outcome on the patient. According to our search, there is a previous case report published in 2020 that shares multiple similarities with our case. Further studies on cupping therapy and its efficacy, mechanism of action, and complications on a larger scale are advised.

## Introduction

Meniere's disease (MD) is a group of disorders characterized by three main symptoms: sensorineural hearing loss, tinnitus, and vertigo [1]. Its underlying cause is still unknown, and symptoms typically begin in adulthood [2]. Over time, hearing often deteriorates gradually, and balance function may gradually decline as well, resulting in persistent dizziness [2]. MD's hallmark is endolymphatic hydrops, and its lifetime prevalence in the United States is 190 per 100,000 people [3]. Meniere's syndrome is a similar condition but secondary to other etiologies like viral infections or trauma [4]. MD is usually chronic, manifesting in one or more rarely both ears, with remissions lasting several months to years [5]. Although MD is known to be idiopathic, viral infections (specifically herpes simplex virus (HSV) types 1 and 2, varicella-zoster virus, and cytomegalovirus) and autoimmune disorders are thought to be the causes of it [6]. Autoimmune disorders are believed to be the cause of about one-third of MD patients [6]. The diagnosis is clinical, but it is important to differentiate it from other causes of vertigo, such as benign positional vertigo or migraine [5]. MD patients often experience a profound impact on their daily activities, with 86% reporting negative work performance and 70% requiring job changes [7]. Although familial aggregation is common in European and Korean cultures, finding familial MD genes is challenging [8]. MD disease poses an economic burden on both the health system and the patient [9].

MD treatment consists of conservative, medical, and surgical treatments [10]. The most cost-effective treatment for vertigo in MD patients involves dietary alterations like sodium and caffeine restriction, which reduce the frequency and severity of attacks, and increasing oral water intake [7]. The first-line treatment for MD is diuretics, which are used to manage endolymphatic hydrops, even though evidence for their effects on MD is lackluster [11]. Betahistine, a popular treatment in Europe, has been shown to reduce vertigo episodes in MD patients, and it is popular in Europe but not FDA-approved in the United States [10]. If conservative medical management fails, a second-line treatment is minimally invasive intratympanic steroid injections, with dexamethasone being more commonly used due to its side effects compared to methylprednisolone [10]. Endolymphatic sac surgery, a controversial treatment, is considered a third-line treatment if medical management fails [12]. It is a very controversial treatment, as several studies suggest that the evidence of its effect on the vertiginous nature of MD is insufficient [12]. However, another study concluded that there is a low level of evidence supporting the surgery having an actual effect [13]. Despite the controversy, it is still considered a third-line treatment if medical management fails [14].

Cupping therapy is a method using plastic, bamboo, or glass cups to suction the skin over acupuncture points, sore spots, or response zones for therapeutic results [15]. It can be done wet or dry, with dry cupping slightly irritating subcutaneous tissues without drawing blood, while wet cupping draws blood from the dermal microcirculation by sucking the lacerated skin [15]. Cupping has been used in East Asian countries and Western countries, particularly for pain-causing illnesses like knee osteoarthritis, neck pain, and low back pain [15]. In the Arabic language, cupping therapy is referred to as "Al-hijamah," which originates from the word "hajm," meaning sucking [16]. The mechanism behind cupping's therapeutic effects is unknown, but some theories suggest hyperemia or hemostasis which results in a therapeutic effect [17]. Other theories suggest that increased blood circulation due to the suction mechanism can lead to more efficient toxin removal [18]. Cupping therapy is a leading traditional practice after spiritual healing and herbal medicine in Saudi Arabia, with wet cupping being the most common type. [19]. Hijamah is a widely performed medical practice in Islamic nations due to its encouragement by the Islamic prophet "Mohammed." In a "Hadith" narrative, the Prophet Mohammed said, "The best medicine you may treat yourselves with is Hijamah." For that, hijamah is considered, for Muslims, "prophetic medicine." This most likely explains the reason behind the spread of this practice [19].

## Case presentation

Symptom course

A 54-year-old male retired officer was complaining of vertigo bouts that started in 1999 and lasted approximately seven years. These fits of vertigo started suddenly and were very severe in nature, leading to very severe fits of nausea, vomiting, and sometimes diarrhea. The frequency of these episodes was every nine months, but with time, they became more frequent: as frequent as daily. These episodes lasted hours or until the patient resorted to some alleviating methods. Those alleviating methods that the patient made use of were "going into very cold places" and "going to sleep," the latter being the more efficient option. Looking to the left, getting hungry, windy weather, and hot weather were known triggers of vertigo for the patient. Because hunger was a trigger, the patient gained weight over the years. Vertigo was associated with a very mild decrease in the function of hearing in the left ear. The patient faced some difficulties hearing low-frequency voices. It was also associated with left-ear roaring tinnitus. The patient reported a few instances where vertigo episodes caused concurrent loss of consciousness that lasted less than 10 seconds.

The patient reported no head injury, headaches, sensation of ear fullness, or falls. The patient denied any fever, night sweats, weight loss, appetite loss, or chills. Although the patient reported no fatigue, he said that there was severe fatigue after the first episode of vertigo nine years ago.

There was no history of any chronic diseases, thyroid diseases, ear-nose-throat diseases, or autoimmune diseases. There was no history of hospitalization or previous surgeries. The patient was not taking any medication. His family had no history of ear, nose, or throat diseases or genetic diseases. There was no similar presentation in the family. The patient was not a smoker. The patient was not a consumer of alcohol or any illicit drugs. He is a retired officer and happily married with six sons. He reports no stress in his life.

Management course

The patient went to multiple hospitals and was diagnosed with MD in all of them. He was counseled with medical treatment, including dietary changes (i.e., decreasing salt and caffeine intake), which he complied with but didn't lead to any change in symptoms. He was prescribed steroids, diuretics, and betahistine with increasing dosages, which didn't result in any symptom relief. The patient was counseled about vestibular neurectomy and its process, benefits, and possible complications, which he opted not to undergo.

**Figure 1:**
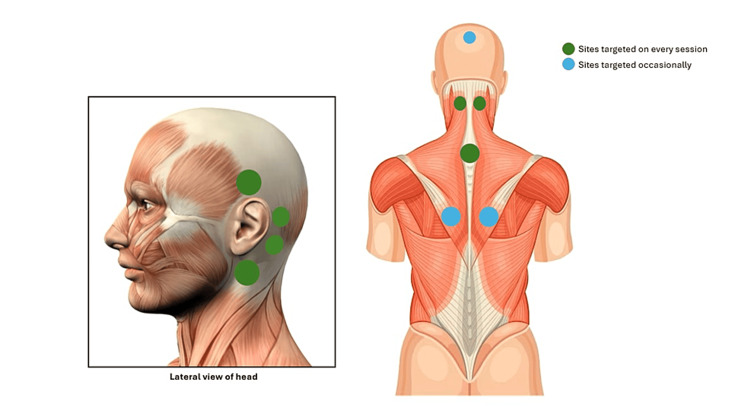
Cupping sites Green circles resemble cupping areas that were targeted in every session, whereas blue circles resemble cupping areas that were targeted occasionally.

The patient was counseled about cupping therapy, which he agreed to. He underwent cupping sessions monthly for two years with a cupping specialist licensed and approved by the Saudi National Center for Complementary and Alternative Medicine (NCCAM). The sessions were conducted either on the 17th, 19th, or 21st of the Hijri month, according to the convenience of the patient, with most of the monthly sessions being on the 17th of the month. When the patient experienced an increase in the severity of symptoms, he conducted two sessions a month, one on the 17th and the next on the second of the next month, with that happening twice during the two-year period. In the last month, the patient conducted three sessions, which were done on the 17th, 19th, and 21st of the same month. The cups were positioned around the left ear, interscapular area, and nape and occasionally on the crown of the head and under the scapula (see Figure 1). The diameter of the cups situated on the interscapular area and under the scapula measured 7 cm. Head and nape cups measured 4.5 cm, and cups situated around the ear measured 3.5 cm. Before commencing cupping sessions, the head was shaved and sterilized, along with the rest of the areas. Afterwards, the skin on the sites of cupping was lacerated with small cuts. After suctioning, the cupping sites were cleaned and sterilized. The estimated blood loss in every session was approximately 150 ml. The patient denied any side effects or skin reactions related to therapy. He has been symptom-free until now for the last 14 years with no history of relapses.

## Discussion

According to our search, there is only one case of this kind reported in 2020, which shares several similarities with this case [20]. The study reports the case of a 48-year-old female, a known case of MD, who presented with sudden unilateral sensorineural hearing loss [20]. She was treated with prednisolone and diuretics [20]. Treatment led to no improvement, similar to the case presented here. Compared to our case, which went for monthly sessions for two years, it was decided that the reported case undergo wet cupping therapy by a professional cupping therapist who applied a light to medium suction pressure for three to five minutes on post-auricular cupping points, followed by superficial scarifications, reapplying, removing, disinfecting, and applying sterile wound dressings. This procedure was repeated for six scheduled sessions, every two weeks [20]. This led to an evident improvement in the patient's condition [20]. In the reported case, the improvement was determined by pure tone audiometry tests [20]. In our case, the improvement was determined subjectively by the patient. As suggested by the aforementioned case, cupping therapy poses an auspicious complementary treatment option. Although the mechanism of action of cupping treatment cannot be explained clearly in our case, we can suggest that hyperemia resulting from cupping therapy may have an effect since vascularity impairments of the endolymphatic sac are noted in MD patients [17,21].

## Conclusions

A case of a 54-year-old male with severe attacks of vertigo who was treated with cupping therapy after conventional medical management was reported in this study. The patient was complaining of bouts of vertigo, which were accompanied by a mild decrease in hearing function. He was started on diet modifications, followed by the addition of diuretics, betahistine, and steroids, all to no avail. The patient was counseled on surgical treatment, which he refused to undergo. He was counseled on undergoing cupping therapy, to which he agreed. The patient discontinued all medications and underwent a two-year period of cupping therapy. Throughout that period, he reported lesser severity and fewer onsets of attacks until ultimately reaching complete remission.

This case suggests that cupping therapy is considered a treatment for MD when medical treatment fails. We encourage further research into the efficacy of this line of therapy on a wider scale. Further studies of the optimum time period and frequency of cupping therapy sessions are also advised. Objective data and measurements are necessary in future studies to determine whether the resolution of symptoms was due to the cupping therapy itself or to the nature of the disease.
